# Crystal structure of poly[di­aqua­(μ_2_-benzene-1,4-di­carboxyl­ato-κ^2^ *O* ^1^:*O* ^4^)(μ_2_-benzene-1,4-di­carboxyl­ato-κ^4^ *O* ^1^,*O* ^1′^:*O* ^4^,*O* ^4′^)bis­(μ_2_-3,3′,5,5′-tetra­methyl-4,4′-bi­pyrazole-κ^2^ *N*:*N*′)dinickel(II)]

**DOI:** 10.1107/S2056989015008415

**Published:** 2015-05-07

**Authors:** Chao Wu, Peng Cao

**Affiliations:** a School of Chemical Engineering and Light Industry, Guangdong University of Technology, Guangzhou 510006, Guangdong, People’s Republic of China

**Keywords:** crystal structure, nickel, coordination polymer, hydrogen bonding

## Abstract

The asymmetric unit of the polymeric title compound, [Ni(C_8_H_4_O_4_)(C_10_H_14_N_4_)(H_2_O)]_*n*_, contains one Ni^2+^ cation, one coordinating water mol­ecule, one 3,3′,5,5′-tetra­methyl-4,4′-bi­pyrazole ligand and half each of two benzene-1,4-di­carboxyl­ate anions, the other halves being generated by inversion symmetry. The Ni^2+^ cation exhibits an octa­hedral N_2_O_4_ coordination sphere defined by the O atoms of the water mol­ecule and two different anions and the N atoms of two symmetry-related *N*-heterocycles. The *N*-heterocycles and both anions bridge adjacent Ni^2+^ cations into a three-dimensional network structure, with one of the anions in a bis-bidentate and the other in a bis-monodentate bridging mode. N—H⋯O and O—H⋯O hydrogen bonds between the N-heterocycles and water mol­ecules as donor groups and the carboxyl­ate O atoms as acceptor groups consolidate the crystal packing.

## Related literature   

In the related water-free coordination polymer {[Zn(tereph­thalate)(H_2_Bpz)]·0.5(H_2_Bpz)}_*n*_ (H_2_Bpz = 3,3′,5,5′-tetra­methyl-4,4′-bi­pyrazole), the Zn^2+^ cation is tetra­hedrally coordinated, see: He *et al.* (2007 ▸). For the structure of [Ni(terephthalate)(pyrazole)_4_], see: Hong *et al.* (2005 ▸).
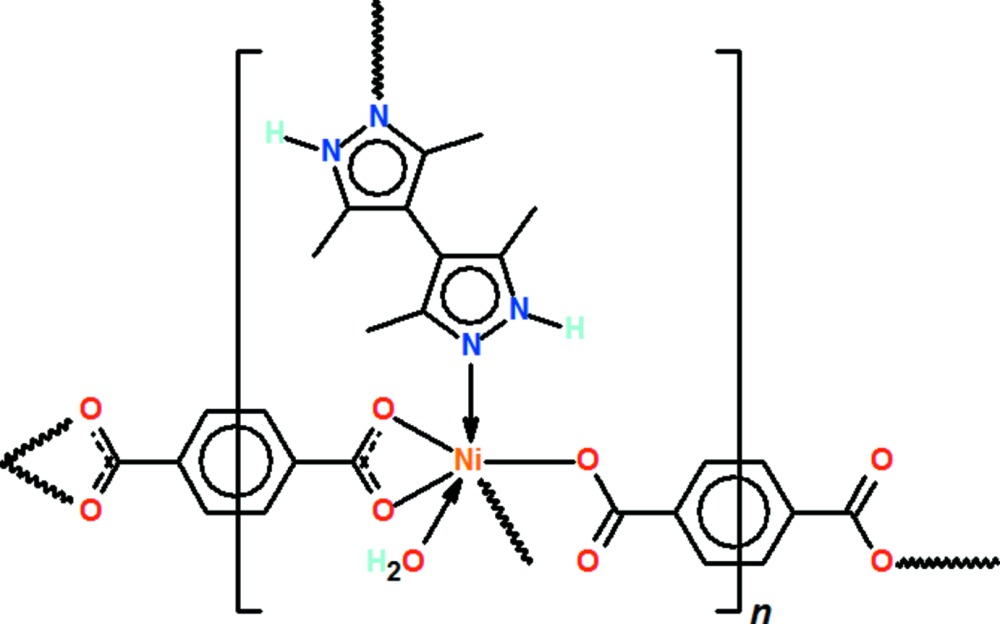


## Experimental   

### Crystal data  

[Ni(C_8_H_4_O_4_)(C_10_H_14_N_4_)(H_2_O)]
*M* *_r_* = 431.09
Monoclinic, 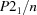

*a* = 11.1603 (7) Å
*b* = 17.3367 (11) Å
*c* = 11.2345 (7) Å
β = 116.081 (1)°
*V* = 1952.3 (2) Å^3^
*Z* = 4
Mo *K*α radiation
μ = 1.03 mm^−1^
*T* = 293 K
0.28 × 0.18 × 0.15 mm

### Data collection  

Bruker SMART diffractometer
Absorption correction: multi-scan (*SADABS*; Bruker, 2014 ▸) *T*_min_ = 0.645, *T*_max_ = 0.746
11758 measured reflections
4236 independent reflections
3627 reflections with *I* > 2σ(*I*)
*R*_int_ = 0.027

### Refinement  

*R*[*F* ^2^ > 2σ(*F* ^2^)] = 0.035
*wR*(*F* ^2^) = 0.098
*S* = 1.04
4236 reflections
272 parameters
4 restraints
H atoms treated by a mixture of independent and constrained refinement
Δρ_max_ = 0.40 e Å^−3^
Δρ_min_ = −0.25 e Å^−3^

### 

Data collection: *APEX2* (Bruker, 2014 ▸); cell refinement: *SAINT* (Bruker, 2014 ▸); data reduction: *SAINT*; program(s) used to solve structure: *SHELXS97* (Sheldrick, 2008 ▸); program(s) used to refine structure: *SHELXL97* (Sheldrick, 2008 ▸); molecular graphics: *X-SEED* (Barbour, 2001 ▸); software used to prepare material for publication: *publCIF* (Westrip, 2010 ▸).

## Supplementary Material

Crystal structure: contains datablock(s) global, I. DOI: 10.1107/S2056989015008415/wm5150sup1.cif

Structure factors: contains datablock(s) I. DOI: 10.1107/S2056989015008415/wm5150Isup2.hkl

**Fig. 1.**
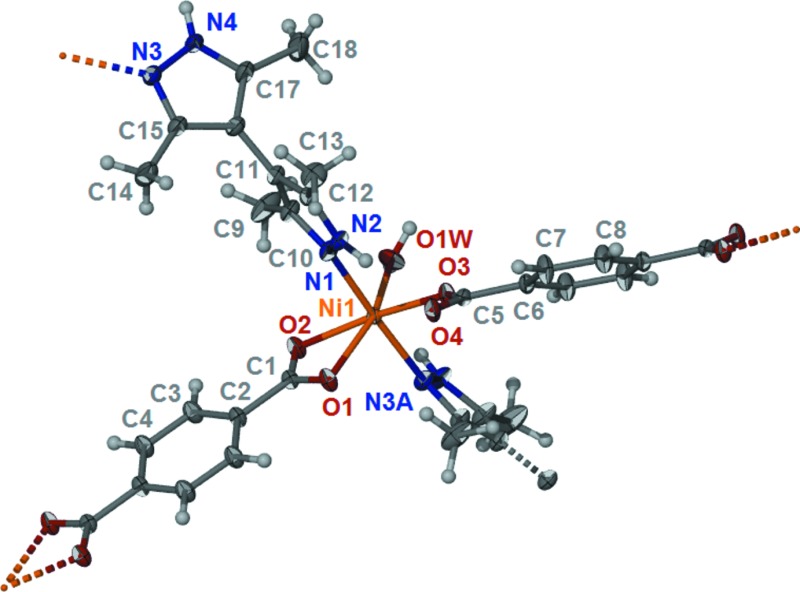
Displacement ellipsoid plot of a fragment of the three-dimensional network of [Ni(H2O)(C10H14N4)(C8H4O4)]n drawn at the 50% probability level. Hydrogen atoms are drawn as spheres of arbitrary radius.

2 10 14 4 8 4 4 n . DOI: 10.1107/S2056989015008415/wm5150fig1.tif
Displacement ellipsoid plot of a fragment of the three-dimensional network of [Ni(H_2_O)(C_10_H_14_N_4_)(C_8_H_4_O_4_)]_*n*_ drawn at the 50% probability level. Hydrogen atoms are drawn as spheres of arbitrary radius.

CCDC reference: 1062289

Additional supporting information:  crystallographic information; 3D view; checkCIF report

## Figures and Tables

**Table 1:** Hydrogen-bond geometry (, )

*D*H*A*	*D*H	H*A*	*D* *A*	*D*H*A*
O1*w*H11O1^i^	0.84(1)	1.80(2)	2.601(2)	159(4)
O1*w*H12O4^i^	0.84(1)	1.90(2)	2.731(2)	173(3)
N2H2O4	0.88(1)	1.98(1)	2.837(2)	164(2)
N4H4O4^ii^	0.87(1)	2.23(1)	3.080(3)	164(2)

Symmetry codes: (i) 
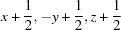
; (ii) 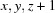.

## supplementary crystallographic information

### S1. Synthesis and crystallization

An aqueous mixture of nickel sulfate hexahydrate (52.6 mg, 0.2 mmol), 3,3',5,5'-tetra­methyl-4,4'-bi­pyrazole (38.1 mg, 0.2 mmol) and 1,4-benzene di­carb­oxy­lic acid (33.2 mg, 0.2 mmol) was placed in a Teflon-lined, stainless-steel reactor. The reactor was heated to 393 K for 48 hours. It was then cooled to room temperatuer at the rate of 5 K per hour. Green crystals were isolated in 70% yield. C, H, N elemental analysis. Calcd. for C_18_H_20_N_4_O_5_Ni: C 50.15, H 4.68, N 13.00%; found C 50.28, H 4.81, N 12.92%.

### S2. Refinement

Carbon-bound H-atoms were placed in calculated positions (C—H 0.93 to 0.96 Å) and were included in the refinement in the riding model approximation, with *U*(H) fixed at 1.2 to 1.5 times *U*_iso_(C). The water and amino H-atoms were located in a difference Fourier map, and were refined with distance restraints of O—H 0.84 (1) Å and N—H 0.88 (1) Å; their *U*_iso_(H) parameter were refined independently.

### Crystal data

**Table:**

[Ni(C_8_H_4_O_4_)(C_10_H_14_N_4_)(H_2_O)]	*F*(000) = 896
*M**_r_* = 431.09	*D*_x_ = 1.467 Mg m^−^^3^
Monoclinic, *P*2_1_/*n*	Mo *K*α radiation, λ = 0.71073 Å
Hall symbol: -P 2yn	Cell parameters from 3721 reflections
*a* = 11.1603 (7) Å	θ = 2.3–26.7°
*b* = 17.3367 (11) Å	µ = 1.03 mm^−^^1^
*c* = 11.2345 (7) Å	*T* = 293 K
β = 116.081 (1)°	Prism, green
*V* = 1952.3 (2) Å^3^	0.28 × 0.18 × 0.15 mm
*Z* = 4	

### Data collection

**Table:**

Bruker SMART diffractometer	4236 independent reflections
Radiation source: fine-focus sealed tube	3627 reflections with *I* > 2σ(*I*)
Graphite monochromator	*R*_int_ = 0.027
ω scans	θ_max_ = 27.0°, θ_min_ = 2.1°
Absorption correction: multi-scan (*SADABS*; Bruker, 2014)	*h* = −13→14
*T*_min_ = 0.645, *T*_max_ = 0.746	*k* = −21→22
11758 measured reflections	*l* = −10→14

### Refinement

**Table:**

Refinement on *F*^2^	Primary atom site location: structure-invariant direct methods
Least-squares matrix: full	Secondary atom site location: difference Fourier map
*R*[*F*^2^ > 2σ(*F*^2^)] = 0.035	Hydrogen site location: inferred from neighbouring sites
*wR*(*F*^2^) = 0.098	H atoms treated by a mixture of independent and constrained refinement
*S* = 1.04	*w* = 1/[σ^2^(*F*_o_^2^) + (0.0583*P*)^2^ + 0.4664*P*] where *P* = (*F*_o_^2^ + 2*F*_c_^2^)/3
4236 reflections	(Δ/σ)_max_ = 0.001
272 parameters	Δρ_max_ = 0.40 e Å^−^^3^
4 restraints	Δρ_min_ = −0.25 e Å^−^^3^

### Fractional atomic coordinates and isotropic or equivalent isotropic displacement parameters (Å^2^)

**Table:**

	*x*	*y*	*z*	*U*_iso_*/*U*_eq_	
Ni1	0.36576 (2)	0.277787 (13)	0.18220 (2)	0.01814 (10)	
O1	0.19348 (13)	0.32361 (8)	0.02675 (15)	0.0266 (3)	
O2	0.29813 (13)	0.38770 (8)	0.21146 (14)	0.0261 (3)	
O3	0.40583 (14)	0.17246 (8)	0.12360 (15)	0.0279 (3)	
O4	0.21805 (14)	0.11750 (9)	−0.02559 (15)	0.0308 (4)	
O1w	0.52381 (16)	0.27350 (9)	0.36205 (17)	0.0316 (4)	
H11	0.561 (3)	0.2350 (13)	0.408 (3)	0.068 (11)*	
H12	0.580 (2)	0.3083 (13)	0.400 (3)	0.057 (9)*	
N1	0.24869 (18)	0.22143 (9)	0.25933 (18)	0.0253 (4)	
N2	0.17275 (17)	0.15986 (10)	0.19515 (17)	0.0243 (4)	
H2	0.172 (2)	0.1420 (14)	0.1219 (15)	0.034 (7)*	
N3	−0.01548 (17)	0.17170 (10)	0.59896 (18)	0.0269 (4)	
N4	0.10431 (19)	0.13770 (12)	0.67197 (19)	0.0326 (4)	
H4	0.125 (3)	0.1247 (15)	0.7539 (13)	0.044 (8)*	
C1	0.20219 (18)	0.38216 (11)	0.0984 (2)	0.0221 (4)	
C2	0.09733 (19)	0.44350 (11)	0.0473 (2)	0.0234 (4)	
C3	0.0975 (2)	0.50261 (12)	0.1296 (2)	0.0285 (5)	
H3	0.1633	0.5043	0.2167	0.034*	
C4	0.0006 (2)	0.55935 (12)	0.0835 (2)	0.0285 (5)	
H4A	0.0007	0.5990	0.1393	0.034*	
C5	0.3431 (2)	0.12127 (11)	0.0413 (2)	0.0241 (4)	
C6	0.42497 (19)	0.05760 (12)	0.0211 (2)	0.0234 (4)	
C7	0.3661 (2)	−0.01133 (13)	−0.0363 (3)	0.0348 (5)	
H7	0.2760	−0.0193	−0.0602	0.042*	
C8	0.4405 (2)	−0.06880 (13)	−0.0584 (2)	0.0326 (5)	
H8	0.4000	−0.1148	−0.0983	0.039*	
C9	0.2976 (3)	0.29163 (17)	0.4676 (3)	0.0512 (8)	
H9A	0.3063	0.3368	0.4226	0.077*	
H9B	0.3844	0.2748	0.5306	0.077*	
H9C	0.2445	0.3036	0.5131	0.077*	
C10	0.2318 (2)	0.22909 (12)	0.3692 (2)	0.0289 (5)	
C11	0.1447 (2)	0.17158 (12)	0.3746 (2)	0.0262 (4)	
C12	0.1100 (2)	0.12834 (12)	0.2614 (2)	0.0261 (4)	
C13	0.0226 (2)	0.05878 (14)	0.2117 (3)	0.0408 (6)	
H13A	−0.0315	0.0634	0.1180	0.061*	
H13B	−0.0339	0.0549	0.2558	0.061*	
H13C	0.0771	0.0134	0.2296	0.061*	
C14	−0.1337 (3)	0.22052 (18)	0.3690 (3)	0.0508 (8)	
H14A	−0.2020	0.2318	0.3964	0.076*	
H14B	−0.1677	0.1850	0.2960	0.076*	
H14C	−0.1066	0.2673	0.3419	0.076*	
C15	−0.0169 (2)	0.18548 (13)	0.4814 (2)	0.0287 (5)	
C16	0.1032 (2)	0.16093 (13)	0.4805 (2)	0.0278 (5)	
C17	0.1780 (2)	0.13039 (15)	0.6040 (2)	0.0358 (5)	
C18	0.3125 (3)	0.0935 (2)	0.6595 (3)	0.0668 (10)	
H18A	0.3648	0.1117	0.7481	0.100*	
H18B	0.3563	0.1065	0.6053	0.100*	
H18C	0.3029	0.0385	0.6606	0.100*	

### Atomic displacement parameters (Å^2^)

**Table:**

	*U*^11^	*U*^22^	*U*^33^	*U*^12^	*U*^13^	*U*^23^
Ni1	0.01651 (14)	0.01908 (15)	0.01932 (16)	0.00261 (9)	0.00832 (11)	0.00007 (10)
O1	0.0223 (7)	0.0220 (7)	0.0284 (8)	0.0043 (5)	0.0047 (6)	−0.0059 (6)
O2	0.0222 (7)	0.0227 (7)	0.0269 (8)	0.0042 (5)	0.0049 (6)	−0.0023 (6)
O3	0.0311 (8)	0.0217 (7)	0.0331 (9)	0.0056 (6)	0.0161 (7)	−0.0049 (6)
O4	0.0239 (7)	0.0372 (9)	0.0292 (9)	0.0117 (6)	0.0097 (7)	−0.0029 (7)
O1w	0.0261 (8)	0.0268 (9)	0.0284 (9)	−0.0014 (6)	−0.0006 (7)	0.0042 (7)
N1	0.0289 (9)	0.0267 (9)	0.0251 (9)	−0.0038 (7)	0.0162 (8)	−0.0052 (7)
N2	0.0255 (8)	0.0302 (9)	0.0211 (9)	−0.0027 (7)	0.0138 (8)	−0.0041 (7)
N3	0.0248 (9)	0.0342 (10)	0.0237 (9)	0.0024 (7)	0.0126 (8)	0.0008 (8)
N4	0.0358 (10)	0.0439 (11)	0.0248 (11)	0.0133 (8)	0.0195 (9)	0.0087 (9)
C1	0.0194 (9)	0.0201 (10)	0.0258 (11)	0.0013 (7)	0.0090 (8)	0.0001 (8)
C2	0.0191 (9)	0.0208 (10)	0.0276 (11)	0.0029 (7)	0.0076 (8)	0.0001 (8)
C3	0.0241 (10)	0.0278 (11)	0.0249 (11)	0.0054 (8)	0.0027 (9)	−0.0024 (9)
C4	0.0295 (11)	0.0234 (10)	0.0265 (12)	0.0061 (8)	0.0069 (9)	−0.0043 (9)
C5	0.0293 (10)	0.0245 (10)	0.0231 (11)	0.0078 (8)	0.0158 (9)	0.0036 (8)
C6	0.0241 (10)	0.0238 (10)	0.0228 (11)	0.0064 (7)	0.0108 (8)	−0.0019 (8)
C7	0.0207 (10)	0.0328 (12)	0.0516 (15)	0.0008 (8)	0.0166 (10)	−0.0108 (11)
C8	0.0273 (11)	0.0262 (11)	0.0442 (14)	−0.0023 (8)	0.0157 (10)	−0.0132 (10)
C9	0.0678 (19)	0.0585 (17)	0.0416 (16)	−0.0270 (15)	0.0372 (15)	−0.0238 (13)
C10	0.0310 (11)	0.0344 (12)	0.0278 (12)	−0.0031 (9)	0.0190 (10)	−0.0045 (9)
C11	0.0266 (10)	0.0320 (11)	0.0251 (11)	0.0031 (8)	0.0159 (9)	0.0009 (9)
C12	0.0238 (10)	0.0306 (11)	0.0268 (11)	0.0009 (8)	0.0138 (9)	0.0009 (9)
C13	0.0405 (13)	0.0400 (14)	0.0455 (15)	−0.0118 (11)	0.0223 (12)	−0.0045 (11)
C14	0.0406 (15)	0.086 (2)	0.0278 (14)	0.0220 (14)	0.0174 (12)	0.0123 (14)
C15	0.0309 (11)	0.0345 (12)	0.0264 (12)	0.0011 (9)	0.0178 (10)	0.0006 (9)
C16	0.0293 (10)	0.0336 (12)	0.0261 (11)	0.0002 (9)	0.0173 (9)	0.0014 (9)
C17	0.0355 (12)	0.0491 (14)	0.0298 (13)	0.0106 (10)	0.0209 (11)	0.0032 (11)
C18	0.0548 (17)	0.109 (3)	0.0436 (17)	0.0446 (19)	0.0283 (14)	0.0177 (18)

### Geometric parameters (Å, º)

**Table:**

Ni1—O1	2.1036 (14)	C5—C6	1.512 (3)
Ni1—O2	2.1276 (14)	C6—C7	1.380 (3)
Ni1—O3	2.0559 (14)	C6—C8^iv^	1.383 (3)
Ni1—O1w	2.0164 (16)	C7—C8	1.388 (3)
Ni1—N1	2.0995 (17)	C7—H7	0.9300
Ni1—N3^i^	2.1185 (17)	C8—C6^iv^	1.383 (3)
O1—C1	1.273 (2)	C8—H8	0.9300
O2—C1	1.254 (2)	C9—C10	1.491 (3)
O3—C5	1.251 (3)	C9—H9A	0.9600
O4—C5	1.262 (2)	C9—H9B	0.9600
O1w—H11	0.836 (10)	C9—H9C	0.9600
O1w—H12	0.836 (10)	C10—C11	1.412 (3)
N1—C10	1.335 (3)	C11—C12	1.378 (3)
N1—N2	1.357 (2)	C11—C16	1.466 (3)
N2—C12	1.342 (3)	C12—C13	1.496 (3)
N2—H2	0.877 (10)	C13—H13A	0.9600
N3—C15	1.335 (3)	C13—H13B	0.9600
N3—N4	1.358 (2)	C13—H13C	0.9600
N3—Ni1^ii^	2.1185 (17)	C14—C15	1.489 (3)
N4—C17	1.352 (3)	C14—H14A	0.9600
N4—H4	0.874 (10)	C14—H14B	0.9600
C1—C2	1.497 (3)	C14—H14C	0.9600
C2—C3	1.380 (3)	C15—C16	1.410 (3)
C2—C4^iii^	1.395 (3)	C16—C17	1.372 (3)
C3—C4	1.383 (3)	C17—C18	1.493 (3)
C3—H3	0.9300	C18—H18A	0.9600
C4—C2^iii^	1.395 (3)	C18—H18B	0.9600
C4—H4A	0.9300	C18—H18C	0.9600
			
O1w—Ni1—O3	94.04 (6)	C8^iv^—C6—C5	119.95 (19)
O1w—Ni1—N1	89.90 (7)	C6—C7—C8	120.42 (19)
O3—Ni1—N1	88.93 (6)	C6—C7—H7	119.8
O1w—Ni1—O1	157.43 (6)	C8—C7—H7	119.8
O3—Ni1—O1	108.52 (6)	C6^iv^—C8—C7	120.0 (2)
N1—Ni1—O1	90.63 (7)	C6^iv^—C8—H8	120.0
O1w—Ni1—N3^i^	90.92 (7)	C7—C8—H8	120.0
O3—Ni1—N3^i^	87.68 (6)	C10—C9—H9A	109.5
N1—Ni1—N3^i^	176.55 (7)	C10—C9—H9B	109.5
O1—Ni1—N3^i^	89.90 (6)	H9A—C9—H9B	109.5
O1w—Ni1—O2	95.26 (6)	C10—C9—H9C	109.5
O3—Ni1—O2	170.69 (6)	H9A—C9—H9C	109.5
N1—Ni1—O2	91.47 (6)	H9B—C9—H9C	109.5
O1—Ni1—O2	62.17 (5)	N1—C10—C11	110.44 (19)
N3^i^—Ni1—O2	91.79 (6)	N1—C10—C9	122.3 (2)
C1—O1—Ni1	89.33 (11)	C11—C10—C9	127.3 (2)
C1—O2—Ni1	88.74 (11)	C12—C11—C10	105.30 (18)
C5—O3—Ni1	137.48 (13)	C12—C11—C16	128.3 (2)
Ni1—O1w—H11	129 (2)	C10—C11—C16	126.3 (2)
Ni1—O1w—H12	127 (2)	N2—C12—C11	106.69 (18)
H11—O1w—H12	102 (3)	N2—C12—C13	122.4 (2)
C10—N1—N2	105.09 (17)	C11—C12—C13	130.9 (2)
C10—N1—Ni1	134.60 (15)	C12—C13—H13A	109.5
N2—N1—Ni1	120.24 (13)	C12—C13—H13B	109.5
C12—N2—N1	112.47 (17)	H13A—C13—H13B	109.5
C12—N2—H2	126.0 (16)	C12—C13—H13C	109.5
N1—N2—H2	121.4 (16)	H13A—C13—H13C	109.5
C15—N3—N4	104.79 (17)	H13B—C13—H13C	109.5
C15—N3—Ni1^ii^	128.69 (15)	C15—C14—H14A	109.5
N4—N3—Ni1^ii^	123.63 (14)	C15—C14—H14B	109.5
C17—N4—N3	112.41 (19)	H14A—C14—H14B	109.5
C17—N4—H4	128.6 (18)	C15—C14—H14C	109.5
N3—N4—H4	119.0 (17)	H14A—C14—H14C	109.5
O2—C1—O1	119.70 (17)	H14B—C14—H14C	109.5
O2—C1—C2	120.65 (18)	N3—C15—C16	110.80 (19)
O1—C1—C2	119.65 (18)	N3—C15—C14	122.5 (2)
C3—C2—C4^iii^	120.16 (18)	C16—C15—C14	126.7 (2)
C3—C2—C1	120.00 (18)	C17—C16—C15	105.50 (19)
C4^iii^—C2—C1	119.84 (18)	C17—C16—C11	126.9 (2)
C2—C3—C4	120.5 (2)	C15—C16—C11	127.4 (2)
C2—C3—H3	119.8	N4—C17—C16	106.5 (2)
C4—C3—H3	119.8	N4—C17—C18	123.6 (2)
C3—C4—C2^iii^	119.38 (19)	C16—C17—C18	129.9 (2)
C3—C4—H4A	120.3	C17—C18—H18A	109.5
C2^iii^—C4—H4A	120.3	C17—C18—H18B	109.5
O3—C5—O4	126.16 (18)	H18A—C18—H18B	109.5
O3—C5—C6	116.72 (18)	C17—C18—H18C	109.5
O4—C5—C6	117.12 (18)	H18A—C18—H18C	109.5
C7—C6—C8^iv^	119.55 (19)	H18B—C18—H18C	109.5
C7—C6—C5	120.49 (18)		
			
O1w—Ni1—O1—C1	−1.3 (2)	Ni1—O3—C5—C6	170.67 (14)
O3—Ni1—O1—C1	178.98 (11)	O3—C5—C6—C7	161.3 (2)
N1—Ni1—O1—C1	89.91 (12)	O4—C5—C6—C7	−18.2 (3)
N3^i^—Ni1—O1—C1	−93.50 (12)	O3—C5—C6—C8^iv^	−19.8 (3)
O2—Ni1—O1—C1	−1.42 (11)	O4—C5—C6—C8^iv^	160.8 (2)
O1w—Ni1—O2—C1	−178.52 (12)	C8^iv^—C6—C7—C8	−0.9 (4)
O3—Ni1—O2—C1	3.8 (4)	C5—C6—C7—C8	178.0 (2)
N1—Ni1—O2—C1	−88.49 (12)	C6—C7—C8—C6^iv^	0.9 (4)
O1—Ni1—O2—C1	1.45 (11)	N2—N1—C10—C11	0.2 (2)
N3^i^—Ni1—O2—C1	90.39 (12)	Ni1—N1—C10—C11	−176.44 (15)
O1w—Ni1—O3—C5	157.3 (2)	N2—N1—C10—C9	−179.1 (2)
N1—Ni1—O3—C5	67.5 (2)	Ni1—N1—C10—C9	4.2 (4)
O1—Ni1—O3—C5	−22.8 (2)	N1—C10—C11—C12	0.2 (3)
N3^i^—Ni1—O3—C5	−112.0 (2)	C9—C10—C11—C12	179.5 (3)
O2—Ni1—O3—C5	−25.1 (5)	N1—C10—C11—C16	179.4 (2)
O1w—Ni1—N1—C10	44.9 (2)	C9—C10—C11—C16	−1.3 (4)
O3—Ni1—N1—C10	138.9 (2)	N1—N2—C12—C11	0.7 (2)
O1—Ni1—N1—C10	−112.6 (2)	N1—N2—C12—C13	−178.74 (19)
N3^i^—Ni1—N1—C10	148.7 (11)	C10—C11—C12—N2	−0.5 (2)
O2—Ni1—N1—C10	−50.4 (2)	C16—C11—C12—N2	−179.7 (2)
O1w—Ni1—N1—N2	−131.40 (15)	C10—C11—C12—C13	178.9 (2)
O3—Ni1—N1—N2	−37.35 (15)	C16—C11—C12—C13	−0.4 (4)
O1—Ni1—N1—N2	71.16 (15)	N4—N3—C15—C16	−0.7 (2)
N3^i^—Ni1—N1—N2	−27.6 (12)	Ni1^ii^—N3—C15—C16	160.26 (15)
O2—Ni1—N1—N2	133.34 (15)	N4—N3—C15—C14	178.0 (2)
C10—N1—N2—C12	−0.6 (2)	Ni1^ii^—N3—C15—C14	−21.1 (3)
Ni1—N1—N2—C12	176.69 (14)	N3—C15—C16—C17	0.7 (3)
C15—N3—N4—C17	0.5 (3)	C14—C15—C16—C17	−177.9 (3)
Ni1^ii^—N3—N4—C17	−161.73 (17)	N3—C15—C16—C11	−174.6 (2)
Ni1—O2—C1—O1	−2.43 (19)	C14—C15—C16—C11	6.8 (4)
Ni1—O2—C1—C2	177.19 (17)	C12—C11—C16—C17	103.9 (3)
Ni1—O1—C1—O2	2.46 (19)	C10—C11—C16—C17	−75.2 (3)
Ni1—O1—C1—C2	−177.17 (17)	C12—C11—C16—C15	−81.9 (3)
O2—C1—C2—C3	−7.0 (3)	C10—C11—C16—C15	99.1 (3)
O1—C1—C2—C3	172.62 (19)	N3—N4—C17—C16	0.0 (3)
O2—C1—C2—C4^iii^	173.1 (2)	N3—N4—C17—C18	−178.6 (3)
O1—C1—C2—C4^iii^	−7.2 (3)	C15—C16—C17—N4	−0.4 (3)
C4^iii^—C2—C3—C4	0.2 (4)	C11—C16—C17—N4	174.9 (2)
C1—C2—C3—C4	−179.6 (2)	C15—C16—C17—C18	178.1 (3)
C2—C3—C4—C2^iii^	−0.2 (4)	C11—C16—C17—C18	−6.6 (5)
Ni1—O3—C5—O4	−9.9 (4)		

Symmetry codes: (i) *x*+1/2, −*y*+1/2, *z*−1/2; (ii) *x*−1/2, −*y*+1/2, *z*+1/2; (iii) −*x*, −*y*+1, −*z*; (iv) −*x*+1, −*y*, −*z*.

### Hydrogen-bond geometry (Å, º)

**Table:**

*D*—H···*A*	*D*—H	H···*A*	*D*···*A*	*D*—H···*A*
O1*w*—H11···O1^v^	0.84 (1)	1.80 (2)	2.601 (2)	159 (4)
O1*w*—H12···O4^v^	0.84 (1)	1.90 (2)	2.731 (2)	173 (3)
N2—H2···O4	0.88 (1)	1.98 (1)	2.837 (2)	164 (2)
N4—H4···O4^vi^	0.87 (1)	2.23 (1)	3.080 (3)	164 (2)

Symmetry codes: (v) *x*+1/2, −*y*+1/2, *z*+1/2; (vi) *x*, *y*, *z*+1.
