# Supplementary figures and images for: Crystal structure of poly[di­aqua­(μ2-benzene-1,4-di­carboxyl­ato-κ2 O 1:O 4)(μ2-benzene-1,4-di­carboxyl­ato-κ4 O 1,O 1′:O 4,O 4′)bis­(μ2-3,3′,5,5′-tetra­methyl-4,4′-bi­pyrazole-κ2 N:N′)dinickel(II)]
Source: Acta Crystallogr E Crystallogr Commun. 2015 May 7;71(Pt 6):m127–8. doi: 10.1107/S2056989015008415 (PMC4459351; doi:10.1107/S2056989015008415)

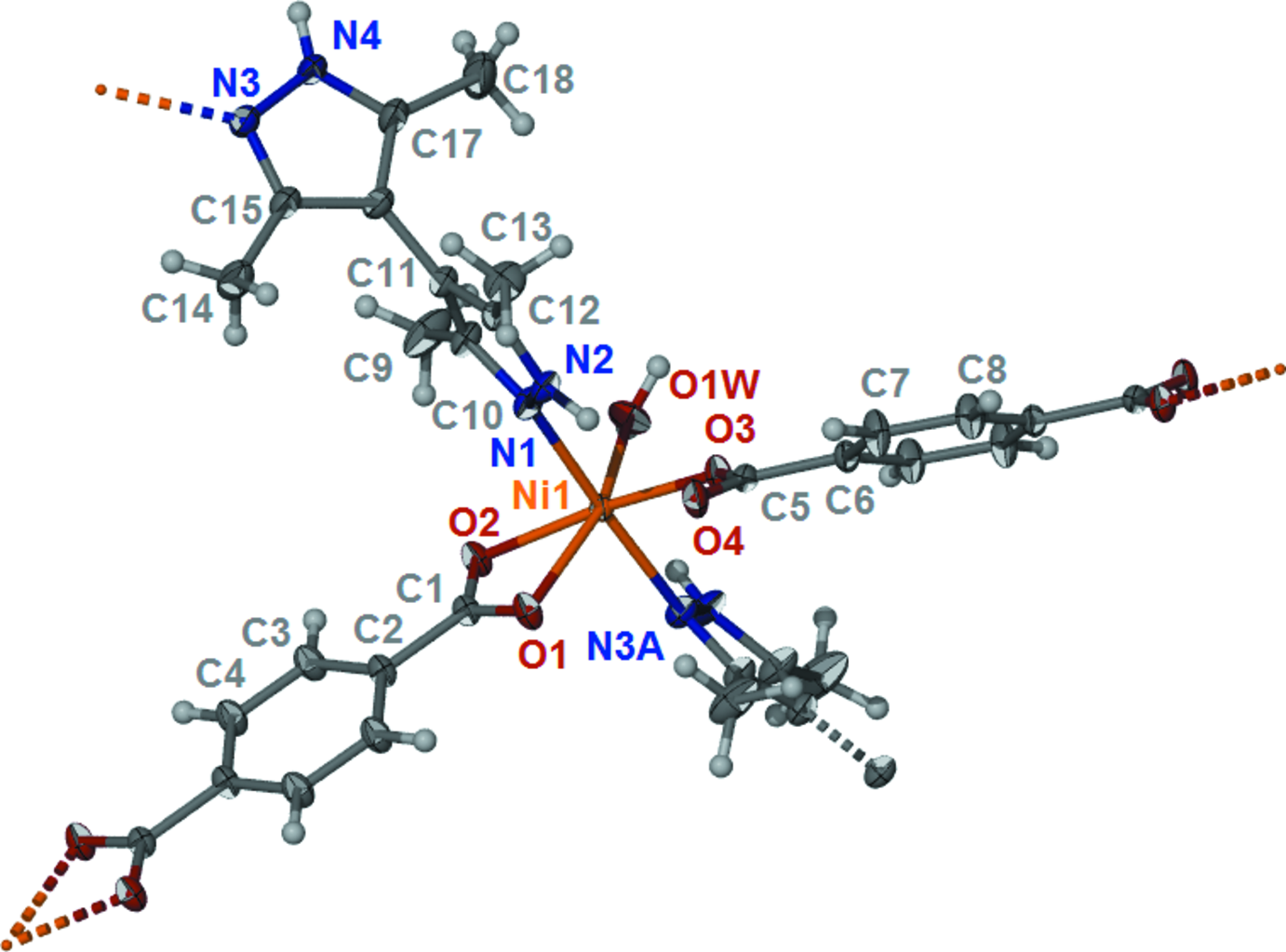

Supplement: Supplementary file 3 [file e-71-0m127-fig1.tif]
